# Supplementary material for: A Splice Variant of NCOR2, BQ323636.1, Confers Chemoresistance in Breast Cancer by Altering the Activity of NRF2
Source: Cancers (Basel). 2020 Feb 26;12(3):533. doi: 10.3390/cancers12030533 (PMC7139508; doi:10.3390/cancers12030533)
Supplement: Supplementary file 1 [file cancers-12-00533-s001.zip › cancers-681551-SI/cancers-681551-Supplmentary Tables.pdf]

Supplementary Materials: A Splice Variant of NCOR2, BQ323636.1, Confers Chemoresistance in Breast Cancer by Altering the Activity of NRF2

Man-Hong Leung, Ho Tsoi, Chun Gong, Ellen PS Man, Stefania Zona, Shang Yao, Eric W.-F. Lam and Ui-Soon Khoo

Supplementary Table S1. RT-PCR Primer sequences used in this study.

Gene of interest	Sequence of the primer (5' to 3')	
**BQ323636.1**	Forward:	AAGGTGGAGCGCATCGAGAAC
	Reverse:	GCATCTGCTTCTCCAGGTTCTCTG
**GAPDH**	Forward:	GAAGGCTGGGGCTCATTT
	Reverse:	CAGGAGGCATTGCTGATGAT
**GCLC**	Forward:	GGATCCTCCAGTTCCTGCAC
	Reverse:	TTCTCCCCAGACAGGACCAA
**GSTP1**	Forward:	CCCTACACCGTGGTCTATTTCC
	Reverse:	CAGGAGGCTTTGAGTGAGC
**HMOX1**	Forward:	AAGACTGCGTTCCTGCTCAAC
	Reverse:	AAAGCCCTACAGCAACTGTCG
**NCOR2**	Forward:	ACGAGGTGTCAGAGATCATCGA
	Reverse:	TGATGAACTTGATGCGCTGCT
**NQO1**	Forward:	GGCAGAAGAGCACTGATCGTA
	Reverse:	TGATGGGATTGAAGTTCATGGC
**NRF2**	Forward:	ATGATGGACTTGGAGCTGCC
	Reverse:	ACTCTTTCCGTCGCTGACTG

Supplementary Table S2. Clinical characteristics of 124 cases of primary breast cancers analysed in this study.

		Number of Cases	Percentage (%)
**Total**		124	
**Median Age**		56	
**T stage**			
	I	14	11.3
	II	22	17.7
	III	5	4.0
	IV	5	4.0
	Missing	78	62.9
**Lymph Node status**			
	Positive	60	40.3
	Negative	50	48.4
	Missing	14	11.3
**Tumor Grade**			
	1	16	12.9
	2	26	21
	3	67	54
	Missing	15	12.1
**Tumor Size**			
	<2cm	14	11.3
	>=2cm	67	54
	Missing	43	34.7
**Estrogen Receptor status**			
	Positive	63	50.8
	Negative	21	16.9
	Missing	40	32.3
**Progesterone receptor status**			
	Positive	40	32.3
	Negative	34	27.4
	Missing	50	40.3
**HER2 receptor status**			
	Positive	31	25.0
	Negative	32	25.8
	Missing	61	49.2
**Triple Negative status**			
	Positive	10	8.1
	Negative	71	57.3
	Missing	43	34.7

Supplementary Table S3. Clinical characteristics of 62 breast cancer patients with record of having received chemotherapeutic treatment analysed in this study.

		Number of Cases	Percentage (%)
**Total**		62	
**Median Age**		47	
**T stage**			
	I	17	27.4
	II	26	41.9
	III	4	6.5
	IV	2	3.2
	Missing	13	21
**Lymph Node status**			
	Positive	51	82.3
	Negative	7	11.3
	Missing	4	6.5
**Tumor Grade**			
	1	2	3.2
	2	17	27.4
	3	39	62.9
	Missing	4	6.5
**Tumor Size**			
	<2cm	8	12.9
	>=2cm	21	33.9
	Missing	33	53.2
**Estrogen Receptor status**			
	Positive	31	48.4
	Negative	21	33.9
	Missing	11	17.7
**Progesterone receptor status**			
	Positive	24	38.7
	Negative	27	43.5
	Missing	11	17.7
**HER2 receptor status**			
	Positive	13	21.0
	Negative	35	56.5
	Missing	14	22.6
**Triple Negative status**			
	Positive	10	16.1
	Negative	39	62.9
	Missing	13	21
